# Supplementary material for: Association of HMGCR rs17671591 and rs3761740 with lipidemia and statin response in Uyghurs and Han Chinese
Source: PeerJ. 2024 Sep 27;12:e18144. doi: 10.7717/peerj.18144 (PMC11441381; doi:10.7717/peerj.18144)

The research methods adopted in the subject

**1. Construction of Blood Lipid Cohort and sample Database of genetic Resources in different ethnic groups**

On the basis of the existing extreme phenotypic cohort of cholesterol and the cohort of people greater than 2 million, the gathering areas of Uygur, Kazak, Tajik and Miao nationalities with ethnic genetic specificity were selected, and 5000 residents of different nationalities over 35 years old were selected by the method of multi-stage cluster sampling. a unified standardized scheme was adopted to carry out epidemiological investigation and cardiovascular event registration, collect biological samples and draw blood lipid profiles of different nationalities.

**2.Establishment of comprehensive risk prediction model of cardiovascular disease in different ethnic groups**

The subject 1 prediction model is verified in different ethnic groups, and a risk prediction model suitable for different ethnic groups is established and compared with the Han population prediction model.

**3. Analysis of genetic factors and different targets affecting hypercholesterolemia and ASCVD risk in different ethnic groups**

Using the strategy of extreme phenotype and pedigree research, the DNA samples were sequenced in full exonome, and the target genes and variation sites were located by bioinformatics analysis, and missense mutations (i.e. changing amino acid sequences) and nonsense mutations (mutations into termination codons) were selected to focus on the study.


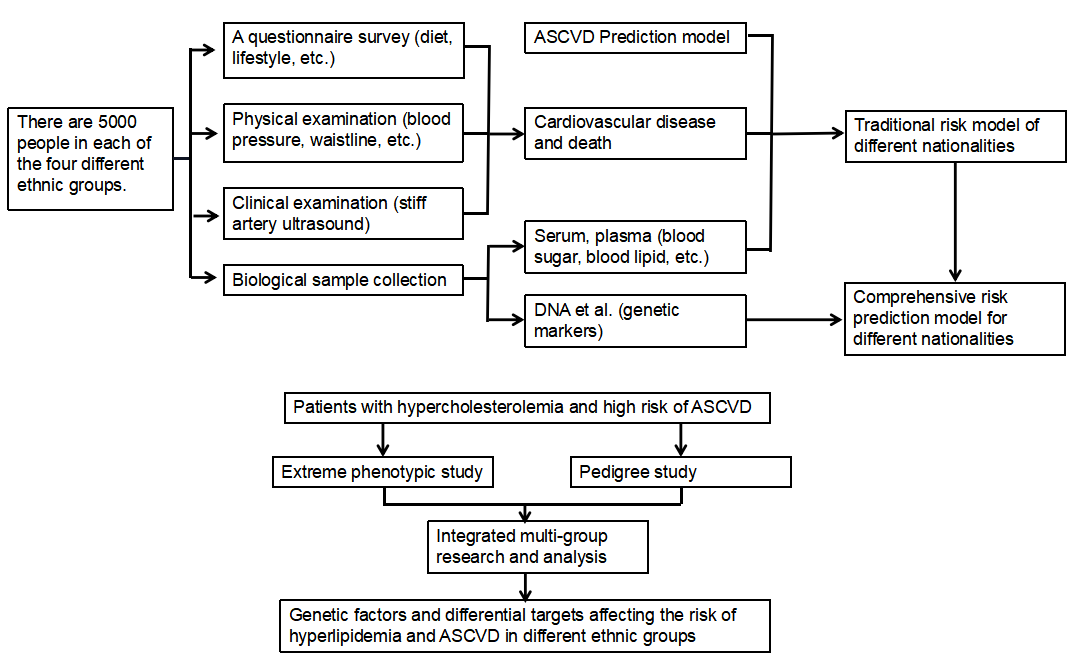

Supplement: Supplemental Information 2 [file peerj-12-18144-s002.docx]
