# Supplementary material for: Association of HMGCR rs17671591 and rs3761740 with lipidemia and statin response in Uyghurs and Han Chinese
Source: PeerJ. 2024 Sep 27;12:e18144. doi: 10.7717/peerj.18144 (PMC11441381; doi:10.7717/peerj.18144)
Supplement: Supplemental Information 3 — Independent sample t test or ANOVA was conducted to generate the P values.The Hardy-Weinberg equilibrium test was performed by Chi-square test, SNP1 genotypes in Han (P = 0.844) and Uyghur (P = 0.906) populations corresponded to Hardy-Weinberg equilibrium.Abbreviation: TC:total cholesterol; TG:triglycerides; HDL-C:high-density lipoprotein cholesterol; LDL-C:low-density lipoprotein cholesterol; APOA1:apolipoprotein A1; APOB:apolipoprotein B,; Lpa:lipoprotein a; ALT:alanine aminotransferase. [file peerj-12-18144-s003.docx]

**Table S1 Association between different models of SNP1 (rs17671591) and blood lipids before oral statin**

|  |  | **Dominant model** | | | **Recessive model** | | | **Additive model** | | | **Allele** | | | **Genotypes** | | | |
| --- | --- | --- | --- | --- | --- | --- | --- | --- | --- | --- | --- | --- | --- | --- | --- | --- | --- |
| **Han** |  | **CC**  **（n=179）** | **TT+CT（n=226）** | **P** | **TT**  **(n=36)** | **CC+CT**  **(n=369)** | **P** | **CT**  **(n=190)** | **CC+TT**  **(n=215)** | **P** | **C**  **（n=548）** | **T**  **（n=262）** | **P** | **CC**  **(n=179)** | **CT**  **(n=190)** | **TT**  **(n=36)** | **P** |
|  | TG  （mmol/L) | 2.240±  1.287 | 2.090±  1.368 | 0.265 | 1.970±  1.039 | 2.170±  1.358 | 0.381 | 2.110±  1.423 | 2.190±  1.251 | 0.541 | 2.190±  1.335 | 2.070±  1.327 | 0.224 | 2.240±  1.287 | 2.110±  1.423 | 1.970±  1.039 | 0.451 |
|  | TC  (mmol/L) | 5.160±  0.891 | 5.130±  0.989 | 0.696 | 4.850±  0.719 | 5.170±  0.961 | 0.053 | 5.180±  1.025 | 5.110±  0.871 | 0.478 | 5.170±  0.938 | 5.090±  0.960 | 0.261 | 5.160±  0.891 | 5.180±  1.025 | 4.850±  0.719 | 0.153 |
|  | HDL-C  (mmol/L) | 1.120±  0.289 | 1.120±  0.364 | 0.998 | 1.100±  0.339 | 1.120±  0.332 | 0.764 | 1.120±  0.370 | 1.120±  0.297 | 0.862 | 1.120±  0.318 | 1.120±  0.360 | 0.899 | 1.120±  0.289 | 1.120±  0.370 | 1.100±  0.339 | 0.952 |
|  | LDL-C  (mmol/L) | 3.470±  0.693 | 3.540±  0.796 | 0.345 | 3.300±  0.632 | 3.530±  0.76 | 0.081 | 3.580±  0.817 | 3.440±  0.684 | 0.053 | 3.510±  0.739 | 3.510±  0.779 | 0.968 | 3.470±  0.693 | 3.580±  0.817 | 3.300±  0.632 | 0.071 |
|  | APOA1  (mmol/L) | 1.250±  0.324 | 1.240±  0.232 | 0.653 | 1.220±  0.219 | 1.240±  0.281 | 0.612 | 1.240±  0.235 | 1.240±  0.309 | 0.874 | 1.240±  0.296 | 1.230±  0.230 | 0.578 | 1.250±  0.324 | 1.240±  0.235 | 1.220±  0.219 | 0.835 |
|  | APOB  (mmol/L) | 1.100±  0.251 | 1.090±  0.256 | 0.927 | 1.040±  0.214 | 1.100±  0.25006 | 0.169 | 1.110±  0.262 | 1.090±  0.246 | 0.488 | 1.100±  0.254 | 1.090±  0.251 | 0.509 | 1.100±  0.251 | 1.110±  0.262 | 1.040±  0.214 | 0.371 |
|  | Lpa  (mg/L) | 238.210±  232.189 | 237.540±  227.710 | 0.977 | 230.180±  225.955 | 238.590±  230.039 | 0.834 | 238.940±  228.612 | 236.860±  230.648 | 0.928 | 238.470±  230.530 | 236.520±  227.050 | 0.91 | 238.21±  232.189 | 238.94±  228.612 | 230.18±  225.955 | 0.381 |
|  | NonHDLC(mmol/L) | 4.047±  0.889 | 4.010±  1.002 | 0.698 | 3.751±  0.762 | 4.053±  0.966 | 0.07 | 4.058±  1.035 | 3.997±  0.874 | 0.52 | 4.051±  0.940 | 3.974±  0.975 | 0.284 | 4.047±  0.889 | 4.058±  1.035 | 3.751±  0.762 | 0.142 |
| **Uyghur** |  | **CC（n=142）** | **TT+CT（n=231）** | **P** | **TT**  **(n=56)** | **CC+CT**  **(n=317)** | **P** | **CT**  **(n=175)** | **CC+TT(n=198)** | **P** | **C**  **（n=459）** | **T**  **（n=287）** | **P** | **CC**  **(n=142)** | **CT**  **(n=175)** | **TT**  **(n=56)** | **P** |
|  | TG  （mmol/L) | 2.330±  1.721 | 2.280±  1.451 | 0.754 | 2.270±  1.206 | 2.300±  1.613 | 0.899 | 2.280±  1.525 | 2.310±  1.590 | 0.831 | 2.310±  1.646 | 2.270±  1.405 | 0.774 | 2.330±  1.721 | 2.280±  1.525 | 2.270±  1.206 | 0.952 |
|  | TC  (mmol/L) | 4.810±  0.873 | 4.970±  1.175 | 0.143 | 5.030±  1.345 | 4.890±  1.017 | 0.47 | 4.960±  1.118 | 4.870±  1.030 | 0.427 | 4.860±  0.974 | 4.980±  1.207 | 0.155 | 4.810±  0.873 | 4.960±  1.118 | 5.030±  1.345 | 0.313 |
|  | HDL-C  (mmol/L) | 0.930±  0.254 | 0.980±  0.254 | 0.05 | 0.980±  0.242 | 0.960±  0.258 | 0.608 | 0.980±  0.259 | 0.940±  0.251 | 0.124 | 0.950±  0.257 | 0.980±  0.252 | 0.099 | 0.930±  0.254 | 0.980±  0.259 | 0.980±  0.242 | 0.145 |
|  | LDL-C  (mmol/L) | 3.220±  0.665 | 3.410±  0.945 | **0.022** | 3.420±  0.973 | 3.330±  0.832 | 0.465 | 3.410±  0.939 | 3.280±  0.767 | 0.126 | 3.300±  0.785 | 3.420±  0.949 | 0.073 | 3.220±  0.665 | 3.410±  0.939 | 3.420±  0.973 | 0.108 |
|  | APOA1  (mmol/L) | 1.100±  0.238 | 1.150±  0.207 | 0.057 | 1.150±  0.202 | 1.130±  0.223 | 0.621 | 1.150±  0.209 | 1.120±  0.228 | 0.135 | 1.120±  0.228 | 1.150±  0.205 | 0.11 | 1.100±  0.238 | 1.150±  0.209 | 1.150±  0.202 | 0.163 |
|  | APOB  (mmol/L) | 1.030±  0.243 | 1.080±  0.280 | 0.127 | 1.090±  0.264 | 1.060±  0.268 | 0.339 | 1.070±  0.285 | 1.050±  0.250 | 0.422 | 1.050±  0.260 | 1.080±  0.277 | 0.115 | 1.030±  0.243 | 1.070±  0.285 | 1.090±  0.264 | 0.277 |
|  | Lpa  (mg/L) | 256.41±  271.035 | 285.66±  299.942 | 0.346 | 264.090±  242.323 | 276.300±  296.966 | 0.773 | 292.510±  316.388 | 258.570±  262.704 | 0.267 | 270.140±  289.028 | 281.470±  289.372 | 0.605 | 256.41±  271.035 | 292.51±  316.388 | 264.09±  242.323 | 0.525 |
|  | NonHDL-C(mmol/L) | 3.878±  0.872 | 3.992±  1.156 | 0.311 | 4.049±  1.276 | 3.931±  1.015 | 0.442 | 3.974±  1.119 | 3.926±  1.003 | 0.666 | 3.914±  0.973 | 4.003±  1.179 | 0.265 | 3.878±  0.872 | 3.974±  1.119 | 4.049±  1.276 | 0.534 |

Independent sample t test or ANOVA was conducted to generate the P values.The Hardy-Weinberg equilibrium test was performed by Chi-square test, SNP1 genotypes in Han(P=0.844) and Uyghur(P= 0.906) populations corresponded to Hardy-Weinberg equilibrium.

Abbreviation: TC:total cholesterol; TG:triglycerides; HDL-C:high-density lipoprotein cholesterol; LDL-C:low-density lipoprotein cholesterol; APOA1:apolipoprotein A1; APOB:apolipoprotein B,; Lpa:lipoprotein a; ALT:alanine aminotransferase.
