# Supplementary material for: Association of HMGCR rs17671591 and rs3761740 with lipidemia and statin response in Uyghurs and Han Chinese
Source: PeerJ. 2024 Sep 27;12:e18144. doi: 10.7717/peerj.18144 (PMC11441381; doi:10.7717/peerj.18144)
Supplement: Supplemental Information 4 — Independent sample t test or ANOVA was conducted to generate the P values.The Hardy-Weinberg equilibrium test was performed by Chi-square test, SNP1 genotypes in Han(P=0.844) and Uyghur(P= 0.906) populations corresponded to Hardy-Weinberg equilibrium.Abbreviation: TC:total cholesterol; TG:triglycerides; HDL-C:high-density lipoprotein cholesterol; LDL-C:low-density lipoprotein cholesterol; APOA1:apolipoprotein A1; APOB:apolipoprotein B,; Lpa:lipoprotein a; ALT:alanine aminotransferase. [file peerj-12-18144-s004.docx]

**Table S2 Association between different models of SNP1 (rs17671591) and blood lipids after oral statin**

|  |  | **Dominant model** | | | | **Recessive model** | | | | **Additive model** | | | | **Allele** | | | **Genotypes** | | | |
| --- | --- | --- | --- | --- | --- | --- | --- | --- | --- | --- | --- | --- | --- | --- | --- | --- | --- | --- | --- | --- |
| **HAN** |  | **CC**  **(n=179)** | **TT+CT**  **(n=226)** | **P** | **TT**  **(n=36)** | | **TT+CT**  **(n=369)** | **P** | **CT**  **(n=190)** | | **CC+TT**  **(n=215)** | **P** | **C**  **(n=548)** | | **T**  **(n=262)** | **P** | **CC**  **(n=179)** | **CT**  **(n=190)** | **TT** | **P** |
|  | TG  （mmol/L) | 1.921±1.313 | 1.861±1.372 | 0.658 | 1.9±0.967 | | 1.886±1.378 | 0.954 | 1.854±1.438 | | 1.917±1.26 | 0.636 | 1.898±1.356 | | 1.867±1.323 | 0.758 | 1.921±1.313 | 1.854±1.438 | 1.9±0.967 | 0.891 |
|  | TC  (mmol/L) | 3.871±1.054 | 3.877±1.001 | 0.95 | 3.875±1.06 | | 3.874±1.021 | 0.996 | 3.877±0.992 | | 3.871±1.052 | 0.952 | 3.873±1.031 | | 3.877±1.007 | 0.961 | 3.871±1.054 | 3.877±0.992 | 3.875±1.06 | 0.998 |
|  | HDL-C  (mmol/L) | 1.085±0.292 | 1.084±0.282 | 0.958 | 1.046±0.295 | | 1.088±0.285 | 0.395 | 1.091±0.28 | | 1.079±0.292 | 0.666 | 1.087±0.287 | | 1.079±0.283 | 0.685 | 1.085±0.292 | 1.091±0.28 | 1.046±0.295 | 0.684 |
|  | LDL-C  (mmol/L) | 2.318±0.798 | 2.372±0.819 | 0.509 | 2.362±0.753 | | 2.347±0.816 | 0.91 | 2.373±0.833 | | 2.325±0.789 | 0.553 | 2.337±0.809 | | 2.37±0.809 | 0.586 | 2.318±0.798 | 2.373±0.833 | 2.362±0.753 | 0.802 |
|  | APOA1  (mmol/L) | 1.209±0.253 | 1.156±0.244 | 0.033 | 1.095±0.299 | | 1.187±0.244 | 0.041 | 1.167±0.233 | | 1.191±0.264 | 0.324 | 1.194±0.247 | | 1.148±0.252 | 0.014 | 1.209±0.253 | 1.167±0.233 | 1.095±0.299 | 0.032 |
|  | APOB  (mmol/L) | 0.823±0.232 | 0.833±0.265 | 0.704 | 0.839±0.254 | | 0.827±0.251 | 0.791 | 0.831±0.268 | | 0.826±0.235 | 0.817 | 0.826±0.245 | | 0.833±0.263 | 0.691 | 1.097±0.251 | 1.105±0.262 | 1.04±0.214 | 0.371 |
|  | Lpa  (mg/L) | 245.506±246.168 | 244.281±249.937 | 0.961 | 272.127±265.881 | | 242.366±246.511 | 0.51 | 239.393±247.455 | | 249.669±248.882 | 0.68 | 243.393±246.177 | | 247.899±251.694 | 0.811 | 245.506±246.168 | 239.393±247.455 | 272.127±265.881 | 0.783 |
|  | NonHDLC  (mmol/L) | 2.77±1.058 | 2.781±1.049 | 0.915 | 2.829±1.056 | | 2.771±1.052 | 0.751 | 2.772±1.05 | | 2.78±1.055 | 0.94 | 2.77±1.053 | | 2.787±1.048 | 0.829 | 2.77±1.058 | 2.772±1.05 | 2.829±1.056 | 0.951 |
| **Uyghur** |  | **CC（n=142）** | **TT+CT（n=231）** | **P** | **TT(n=56)** | | **TT+CT(n=317)** | **P** | **CT(n=175)** | | **CC+TT(n=198)** | **P** | **C**  **(n=459)** | | **T**  **（n=287）** | **P** | **CC(n=142)** | **CT(n=175)** | **TT(n=56)** | **P** |
|  | TG  (mmol/L) | 2.026±1.661 | 1.921±1.114 | 0.465 | 2.026±1.661 | | 1.921±1.114 | 0.465 | 1.867±1.037 | | 2.044±1.569 | 0.194 | 1.966±1.454 | | 1.954±1.157 | 0.909 | 2.026±1.661 | 1.867±1.037 | 2.09±1.322 | 0.43 |
|  | TC  (mmol/L) | 3.964±0.984 | 4.149±1.239 | 0.111 | 3.964±0.984 | | 4.149±1.239 | 0.111 | 4.21±1.257 | | 3.963±1.036 | 0.039 | 4.057±1.1 | | 4.112±1.225 | 0.536 | 3.964±0.984 | 4.21±1.257 | 3.961±1.169 | 0.118 |
|  | HDL-C  (mmol/L) | 0.963±0.229 | 0.987±0.283 | 0.395 | 0.963±0.229 | | 0.987±0.283 | 0.395 | 0.989±0.281 | | 0.967±0.248 | 0.416 | 0.973±0.249 | | 0.985±0.285 | 0.553 | 0.963±0.229 | 0.989±0.281 | 0.978±0.294 | 0.671 |
|  | LDL-C  (mmol/L) | 2.485±0.815 | 2.704±0.944 | 0.022 | 2.485±0.815 | | 2.704±0.944 | 0.022 | 2.775±0.923 | | 2.485±0.864 | 0.002 | 2.595±0.867 | | 2.662±0.954 | 0.326 | 2.485±0.815 | 2.775±0.923 | 2.486±0.983 | 0.008 |
|  | APOA1  (mmol/L) | 1.082±0.181 | 1.108±0.313 | 0.37 | 1.082±0.181 | | 1.108±0.313 | 0.37 | 1.097±0.215 | | 1.099±0.312 | 0.953 | 1.088±0.194 | | 1.115±0.36 | 0.25 | 1.082±0.181 | 1.097±0.215 | 1.144±0.518 | 0.372 |
|  | APOB  (mmol/L) | 0.862±0.261 | 0.919±0.277 | 0.054 | 0.862±0.261 | | 0.919±0.277 | 0.054 | 0.929±0.278 | | 0.868±0.264 | 0.035 | 0.887±0.269 | | 0.912±0.276 | 0.234 | 0.862±0.261 | 0.929±0.278 | 0.885±0.272 | 0.093 |
|  | Lpa  (mg/L) | 232.187±218.444 | 273.933±302.478 | 0.163 | 232.187±218.444 | | 273.933±302.478 | 0.163 | 281.228±318.446 | | 237.215±225.61 | 0.13 | 250.912±261.713 | | 269.427±292.111 | 0.381 | 232.187±218.444 | 281.228±318.446 | 250.364±245.106 | 0.293 |
|  | NonHDLC  (mmol/L) | 0.042±0.202 | 0.091±0.289 | 0.055 | 0.042±0.202 | | 0.091±0.289 | 0.055 | 0.092±0.29 | | 0.056±0.23 | 0.178 | 3.078±1.079 | | 3.117±1.199 | 0.648 | 3.001±0.99 | 3.202±1.205 | 2.983±1.188 | 0.21 |

Independent sample t test or ANOVA was conducted to generate the P values.The Hardy-Weinberg equilibrium test was performed by Chi-square test, SNP1 genotypes in Han(P=0.844) and Uyghur(P= 0.906) populations corresponded to Hardy-Weinberg equilibrium.

Abbreviation: TC:total cholesterol; TG:triglycerides; HDL-C:high-density lipoprotein cholesterol; LDL-C:low-density lipoprotein cholesterol; APOA1:apolipoprotein A1; APOB:apolipoprotein B,; Lpa:lipoprotein a; ALT:alanine aminotransferase.
