# Supplementary material for: Association of HMGCR rs17671591 and rs3761740 with lipidemia and statin response in Uyghurs and Han Chinese
Source: PeerJ. 2024 Sep 27;12:e18144. doi: 10.7717/peerj.18144 (PMC11441381; doi:10.7717/peerj.18144)
Supplement: Supplemental Information 5 — Chi-square test was conducted to generate the P values.The Hardy-Weinberg equilibrium test was performed by Chi-square test, SNP1 genotypes in Han(P=0.844) and Uyghur(P= 0.906) populations corresponded to Hardy-Weinberg equilibrium.Abbreviation: TC:total cholesterol; TG:triglycerides; HDL-C:high-density lipoprotein cholesterol; LDL-C:low-density lipoprotein cholesterol; APOA1:apolipoprotein A1; APOB:apolipoprotein B,; Lpa:lipoprotein a; ALT:alanine aminotransferase. [file peerj-12-18144-s005.docx]

**Table S3 Association between different models of SNP1 (rs17671591) and the rate of hyperlipidemia before oral statin**

| Ethnic Group |  | Dominant model | | | Recessive model | | | Additive model | | | Allele | | | Genotypes | | | |
| --- | --- | --- | --- | --- | --- | --- | --- | --- | --- | --- | --- | --- | --- | --- | --- | --- | --- |
| Han |  | CC  （n=179） | TT+CT  （n=226） | P | TT  (n=36) | CC+CT  (n=369) | P | CT  (n=190) | CC+TT  (n=215) | P | C  （n=548） | T  （n=262） | P | CC  (n=179) | CT  (n=190) | TT  (n=36) | P |
|  | High TG  (%) | 32.778 | 29.515 | 0.48 | 33.333 | 30.728 | 0.747 | 28.796 | 32.87 | 0.375 | 31.397 | 30.038 | 0.695 | 32.778 | 28.796 | 33.333 | 0.673 |
|  | High TC  (%) | 10.555 | 14.537 | 0.232 | 0 | 14.016 | **0.032** | 17.277 | 8.796 | **0.011** | 12.886 | 12.548 | 0.892 | 10.556 | 17.277 | 0 | **0.008** |
|  | Low HDL-C  (%) | 38.889 | 40.529 | 0.737 | 41.667 | 39.623 | 0.811 | 40.314 | 39.352 | 0.843 | 39.383 | 40.684 | 0.723 | 38.889 | 40.314 | 41.667 | 0.934 |
|  | High LDL-C  (%) | 17.778 | 22.026 | 0.289 | 16.667 | 20.485 | 0.586 | 23.037 | 17.593 | 0.172 | 19.601 | 21.293 | 0.574 | 17.778 | 23.037 | 16.667 | 0.389 |
|  | Low APOA1  (%) | 6.704 | 6.195 | 0.836 | 5.556 | 6.504 | 1 | 6.316 | 6.512 | 0.936 | 6.569 | 6.107 | 0.802 | 6.704 | 6.316 | 5.556 | 0.965 |
|  | High APOB  (%) | 46.369 | 42.92 | 0.488 | 38.889 | 44.986 | 0.482 | 43.684 | 45.116 | 0.772 | 45.438 | 42.366 | 0.411 | 46.369 | 43.684 | 38.889 | 0.683 |
|  | High Lpa  (%) | 23.034 | 21.333 | 0.683 | 22.222 | 22.071 | 0.983 | 21.164 | 22.897 | 0.676 | 22.385 | 21.455 | 0.766 | 23.033 | 21.164 | 22.222 | 0.911 |
|  | High NonHDLC  (%) | 16.111 | 18.943 | 0.457 | 8.333 | 18.598 | 0.123 | 20.942 | 14.815 | 0.106 | 17.786 | 17.49 | 0.918 | 16.111 | 20.942 | 8.333 | 0.145 |
| Uyghur |  | CC  （n=142） | TT+CT  （n=231） | P | TT  (n=56) | CC+CT  (n=317) | P | CT  (n=175) | CC+TT  (n=198) | P | C  （n=459） | T  （n=287） | P | CC  (n=142) | CT  (n=175) | TT  (n=56) | P |
|  | High TG  (%) | 30.282 | 36.364 | 0.229 | 39.286 | 33.123 | 0.37 | 35.429 | 32.828 | 0.597 | 32.244 | 36.934 | 0.188 | 0.303 | 0.354 | 0.393 | 0.421 |
|  | High TC  (%) | 7.042 | 12.554 | 0.091 | 19.643 | 8.833 | **0.015** | 10.286 | 10.606 | 0.92 | 8.279 | 13.937 | **0.014** | 0.07 | 0.103 | 0.196 | **0.033** |
|  | Low HDL-C  (%) | 72.535 | 54.545 | **0.001** | 55.357 | 62.461 | 0.314 | 54.286 | 67.677 | **0.008** | 65.577 | 54.704 | **0.003** | 0.725 | 0.543 | 0.554 | **0.002** |
|  | High LDL-C  (%) | 7.746 | 14.719 | **0.045** | 17.857 | 11.041 | 0.149 | 13.714 | 10.606 | 0.358 | 10.022 | 15.331 | **0.03** | 0.077 | 0.137 | 0.179 | 0.095 |
|  | Low APOA1  (%) | 3.546 | 3.057 | 0.796 | 3.636 | 3.175 | 0.858 | 2.874 | 3.571 | 0.705 | 3.289 | 3.169 | 0.928 | 3.546 | 2.876 | 3.636 | 0.93 |
|  | High APOB  (%) | 39.716 | 40.611 | 0.865 | 40 | 40.317 | 0.965 | 40.805 | 39.796 | 0.843 | 40.132 | 40.493 | 0.922 | 39.716 | 40.805 | 40 | 0.98 |
|  | High Lpa  (%) | 25.532 | 30.702 | 0.286 | 30.909 | 28.343 | 0.698 | 30.636 | 27.041 | 0.446 | 27.412 | 30.634 | 0.346 | 25.532 | 30.636 | 30.909 | 0.566 |
|  | High NonHDLC  (%) | 11.972 | 16.017 | 0.281 | 25 | 12.618 | **0.015** | 13.143 | 15.657 | 0.491 | 12.418 | 17.77 | **0.043** | 0.12 | 0.131 | 0.25 | **0.05** |

Chi-square test was conducted to generate the P values.The Hardy-Weinberg equilibrium test was performed by Chi-square test, SNP1 genotypes in Han(P=0.844) and Uyghur(P= 0.906) populations corresponded to Hardy-Weinberg equilibrium.

Abbreviation: TC:total cholesterol; TG:triglycerides; HDL-C:high-density lipoprotein cholesterol; LDL-C:low-density lipoprotein cholesterol; APOA1:apolipoprotein A1; APOB:apolipoprotein B,; Lpa:lipoprotein a; ALT:alanine aminotransferase.
