# Supplementary material for: Association of HMGCR rs17671591 and rs3761740 with lipidemia and statin response in Uyghurs and Han Chinese
Source: PeerJ. 2024 Sep 27;12:e18144. doi: 10.7717/peerj.18144 (PMC11441381; doi:10.7717/peerj.18144)
Supplement: Supplemental Information 6 — Chi-square test was conducted to generate the P values.The Hardy-Weinberg equilibrium test was performed by Chi-square test, SNP1 genotypes in Han(P=0.844) and Uyghur(P= 0.906) populations corresponded to Hardy-Weinberg equilibrium.Abbreviation: TC:total cholesterol; TG:triglycerides; HDL-C:high-density lipoprotein cholesterol; LDL-C:low-density lipoprotein cholesterol; APOA1:apolipoprotein A1; APOB:apolipoprotein B,; Lpa:lipoprotein a; ALT:alanine aminotransferase. [file peerj-12-18144-s006.docx]

**Table S4 Association between different models of SNP1 (rs17671591) and the rate of hyperlipidemia after oral statin**

|  |  | Dominant model | | | | Recessive model | | | | Additive model | | | | Allele | | | Genotypes | | | |
| --- | --- | --- | --- | --- | --- | --- | --- | --- | --- | --- | --- | --- | --- | --- | --- | --- | --- | --- | --- | --- |
| HAN |  | CC  （n=179） | TT+CT  (n=226) | P | TT  (n=36) | | TT+CT  (n=369) | P | CT  (n=190) | | CC+TT  (n=215) | P | C  (n=548) | | T  (n=262) | P | CC  (n=179) | CT  (n=190) | TT | P |
|  | High TG(%) | 26.257 | 19.912 | 0.130 | 25.000 | | 22.493 | 0.732 | 18.947 | | 26.047 | 0.089 | 23.723 | | 20.611 | 0.323 | 26.257 | 18.947 | 25.000 | 0.232 |
|  | High TC(%) | 4.469 | 3.097 | 0.468 | 2.778 | | 3.794 | 0.758 | 3.158 | | 4.186 | 0.585 | 4.015 | | 3.053 | 0.498 | 4.469 | 3.158 | 2.778 | 0.764 |
|  | Low HDL-C(%) | 44.693 | 46.460 | 0.723 | 58.333 | | 44.444 | 0.110 | 44.211 | | 46.977 | 0.577 | 44.526 | | 48.092 | 0.341 | 44.693 | 44.211 | 58.333 | 0.278 |
|  | High LDL-C(%) | 3.911 | 2.655 | 0.476 | 2.778 | | 3.252 | 0.878 | 2.632 | | 3.721 | 0.535 | 3.467 | | 2.672 | 0.548 | 3.911 | 2.632 | 2.778 | 0.775 |
|  | Low APOA1(%) | 47.191 | 58.371 | 0.026 | 69.697 | | 51.913 | 0.050 | 56.383 | | 50.711 | 0.257 | 50.368 | | 59.843 | 0.012 | 47.191 | 56.383 | 69.697 | 0.031 |
|  | High APOB(%) | 11.798 | 13.122 | 0.691 | 15.152 | | 12.295 | 0.635 | 12.766 | | 12.322 | 0.894 | 12.132 | | 13.386 | 0.618 | 11.798 | 12.766 | 15.152 | 0.859 |
|  | High Lpa(%) | 25.281 | 24.887 | 0.928 | 33.333 | | 24.317 | 0.252 | 23.404 | | 26.540 | 0.471 | 24.632 | | 25.984 | 0.681 | 25.281 | 23.404 | 33.333 | 0.477 |
|  | High NonHDLC(%) | 5.587 | 4.867 | 0.746 | 2.778 | | 5.420 | 0.495 | 5.263 | | 5.116 | 0.947 | 5.474 | | 4.580 | 0.591 | 5.587 | 5.263 | 2.778 | 0.785 |
| Uyghur |  | CC  （n=142） | TT+CT  (n=231) | P | TT  (n=56) | | TT+CT  (n=317) | P | CT  (n=175) | | CC+TT  (n=198) | P | C  (n=459) | | T  (n=287) | P | CC  (n=142) | CT  (n=175) | TT  (n=56) | P |
|  | High TG(%) | 26.761 | 24.783 | 0.671 | 30.357 | | 24.684 | 0.370 | 22.989 | | 27.778 | 0.291 | 25.328 | | 25.874 | 0.868 | 26.761 | 22.989 | 30.357 | 0.499 |
|  | High TC(%) | 4.225 | 5.652 | 0.544 | 8.929 | | 4.430 | 0.159 | 4.598 | | 5.556 | 0.675 | 4.367 | | 6.294 | 0.245 | 4.225 | 4.598 | 8.929 | 0.366 |
|  | Low HDL-C(%) | 61.268 | 59.130 | 0.683 | 51.786 | | 61.392 | 0.176 | 61.494 | | 58.586 | 0.568 | 61.354 | | 57.692 | 0.321 | 61.268 | 61.494 | 51.786 | 0.400 |
|  | High LDL-C(%) | 2.113 | 8.696 | 0.010 | 8.929 | | 5.696 | 0.355 | 8.621 | | 4.040 | 0.067 | 4.585 | | 8.741 | 0.022 | 2.113 | 8.621 | 8.929 | 0.038 |
|  | Low APOA1(%) | 72.993 | 72.273 | 0.882 | 75.000 | | 72.131 | 0.668 | 71.429 | | 73.545 | 0.655 | 72.398 | | 72.794 | 0.908 | 72.993 | 71.429 | 75.000 | 0.871 |
|  | High APOB(%) | 18.978 | 22.727 | 0.400 | 23.077 | | 20.984 | 0.733 | 22.619 | | 20.106 | 0.563 | 20.362 | | 22.794 | 0.441 | 18.978 | 22.619 | 23.077 | 0.700 |
|  | High Lpa(%) | 25.735 | 28.636 | 0.552 | 23.077 | | 28.289 | 0.437 | 30.357 | | 25.000 | 0.259 | 27.500 | | 27.574 | 0.983 | 25.735 | 30.357 | 23.077 | 0.494 |
|  | High NonHDLC(%) | 4.225 | 9.130 | 0.076 | 8.929 | | 6.962 | 0.601 | 9.195 | | 5.556 | 0.177 | 6.114 | | 9.091 | 0.128 | 4.225 | 9.195 | 8.929 | 0.208 |

Chi-square test was conducted to generate the P values.The Hardy-Weinberg equilibrium test was performed by Chi-square test, SNP1 genotypes in Han(P=0.844) and Uyghur(P= 0.906) populations corresponded to Hardy-Weinberg equilibrium.

Abbreviation: TC:total cholesterol; TG:triglycerides; HDL-C:high-density lipoprotein cholesterol; LDL-C:low-density lipoprotein cholesterol; APOA1:apolipoprotein A1; APOB:apolipoprotein B,; Lpa:lipoprotein a; ALT:alanine aminotransferase.
