# Supplementary material for: Association of HMGCR rs17671591 and rs3761740 with lipidemia and statin response in Uyghurs and Han Chinese
Source: PeerJ. 2024 Sep 27;12:e18144. doi: 10.7717/peerj.18144 (PMC11441381; doi:10.7717/peerj.18144)
Supplement: Supplemental Information 7 — Independent sample t test or ANOVA was conducted to generate the P values.The Hardy-Weinberg equilibrium test was performed by Chi-square test, SNP2 genotypes in Han(P=0.844) and Uyghur(P= 0.534) populations corresponded to Hardy-Weinberg equilibrium.Abbreviation: TC:total cholesterol; TG:triglycerides; HDL-C:high-density lipoprotein cholesterol; LDL-C:low-density lipoprotein cholesterol; APOA1:apolipoprotein A1; APOB:apolipoprotein B,; Lpa:lipoprotein a; ALT:alanine aminotransferase. [file peerj-12-18144-s007.docx]

**Table S5 Association between different models of SNP2(rs3761740) and blood lipids before oral statin**

| **Ethnic Group** |  | **Genotypes** | | | **Allele** | | | | **Additive model** | | |
| --- | --- | --- | --- | --- | --- | --- | --- | --- | --- | --- | --- |
| **Han** |  | **CC(n=380)** | **CA(n=25)** | **P** | **C（n=785）** | **A（n=25）** | **P** | **_** | | **_** | **_** |
|  | TG（mmol/L) | 2.160±1.344 | 2.090±1.182 | 0.788 | 2.160±1.338 | 2.090±1.182 | 0.791 | _ | | _ | _ |
|  | TC(mmol/L) | 5.150±0.941 | 5.060±1.027 | 0.656 | 5.150±0.943 | 5.060±1.027 | 0.66 | _ | | _ | _ |
|  | HDL-C(mmol/L) | 1.120±0.337 | 1.080±0.265 | 0.558 | 1.120±0.334 | 1.080±0.265 | 0.564 | _ | | _ | _ |
|  | LDL-C(mmol/L) | 3.510±0.752 | 3.500±0.762 | 0.987 | 3.510±0.752 | 3.500±0.762 | 0.987 | _ | | _ | _ |
|  | APOA1(mmol/L) | 1.240±0.276 | 1.250±0.278 | 0.923 | 1.240±0.276 | 1.250±0.278 | 0.924 | _ | | _ | _ |
|  | APOB(mmol/L) | 1.100±0.254 | 1.040±0.241 | 0.259 | 1.100±0.253 | 1.040±0.241 | 0.267 | _ | | _ | _ |
|  | Lpa（mg/L) | 235.930±230.832 | 266.660±208.891 | 0.517 | 236.910±229.955 | 266.660±208.891 | 0.523 | _ | | _ | _ |
|  | Non-HDL-C(mmol/L) | 4.029±0.95014 | 3.982±1.006 | 0.812 | 4.027±0.951 | 3.982±1.006 | 0.814 | _ | | _ | _ |
| **Uyhhur** |  | **CC(n=326)** | **AA+CA(n=47)** | **P** | **C（n=696）** | **A（n=50）** | **P** | **CA(n=44)** | | **AA+CC(n=329)** | **P** |
|  | TG（mmol/L) | 2.300±1.591 | 2.270±1.314 | 0.894 | 2.300±1.574 | 2.300±1.303 | 0.997 | 2.230±1.327 | | 2.300±1.588 | 0.779 |
|  | TC(mmol/L) | 4.850±0.939 | 5.350±1.684 | 0.053 | 4.880±1.013 | 5.280±1.655 | 0.103 | 5.420±1.712 | | 4.840±0.937 | **0.032** |
|  | HDL-C(mmol/L) | 0.960±0.257 | 0.980±0.247 | 0.699 | 0.960±0.256 | 0.970±0.243 | 0.855 | 0.980±0.251 | | 0.960±0.256 | 0.551 |
|  | LDL-C(mmol/L) | 3.290±0.726 | 3.720±1.415 | **0.043** | 3.320±0.797 | 3.670±1.39 | 0.086 | 3.790±1.44 | | 3.280±0.724 | **0.027** |
|  | APOA1(mmol/L) | 1.130±0.221 | 1.140±0.211 | 0.685 | 1.130±0.22 | 1.130±0.21 | 0.97 | 1.160±0.211 | | 1.130±0.221 | 0.427 |
|  | APOB(mmol/L) | 1.050±0.231 | 1.160±0.433 | 0.097 | 1.050±0.251 | 1.150±0.421 | 0.126 | 1.160±0.446 | | 1.050±0.23 | 0.091 |
|  | Lpa（mg/L) | 264.200±276.643 | 344.910±359.567 | 0.074 | 269.600±283.568 | 341.720±352.170 | 0.088 | 348.530±367.781 | | 264.460±276.054 | 0.07 |
|  | Non-HDL-C(mmol/L) | 3.888±0.9354 | 4.370±1.62927 | 0.053 | 3.923±1.003 | 4.309±1.600 | 0.098 | 4.439±1.65935 | | 3.883±0.933 | **0.034** |

Independent sample t test or ANOVA was conducted to generate the P values.The Hardy-Weinberg equilibrium test was performed by Chi-square test, SNP2 genotypes in Han(P=0.844) and Uyghur(P= 0.534) populations corresponded to Hardy-Weinberg equilibrium.

Abbreviation: TC:total cholesterol; TG:triglycerides; HDL-C:high-density lipoprotein cholesterol; LDL-C:low-density lipoprotein cholesterol; APOA1:apolipoprotein A1; APOB:apolipoprotein B,; Lpa:lipoprotein a; ALT:alanine aminotransferase.
