# Supplementary material for: Association of HMGCR rs17671591 and rs3761740 with lipidemia and statin response in Uyghurs and Han Chinese
Source: PeerJ. 2024 Sep 27;12:e18144. doi: 10.7717/peerj.18144 (PMC11441381; doi:10.7717/peerj.18144)
Supplement: Supplemental Information 8 — Independent sample t test or ANOVA was conducted to generate the P values.The Hardy-Weinberg equilibrium test was performed by Chi-square test, SNP2 genotypes in Han(P=0.844) and Uyghur(P= 0.534) populations corresponded to Hardy-Weinberg equilibrium.Abbreviation: TC:total cholesterol; TG:triglycerides; HDL-C:high-density lipoprotein cholesterol; LDL-C:low-density lipoprotein cholesterol; APOA1:apolipoprotein A1; APOB:apolipoprotein B,; Lpa:lipoprotein a; ALT:alanine aminotransferase. [file peerj-12-18144-s008.docx]

**Table S6 Association between different models of SNP2(rs3761740) and blood lipids after oral statin**

| **Ethnic Group** |  | **Genotypes** | | | **Allele** | | | **Additive model** | | |
| --- | --- | --- | --- | --- | --- | --- | --- | --- | --- | --- |
| **Han** |  | **CC(n=380)** | **CA(n=25)** | **P** | **C（n=785）** | **A（n=25）** | **P** | **_** | **_** | **_** |
|  | **TG（mmol/L)** | **1.89±1.365** | **1.852±1.019** | **0.893** | **1.889±1.354** | **1.852±1.019** | **0.894** | **_** | **_** | **_** |
|  | **TC(mmol/L)** | **3.875±1.03** | **3.865±0.926** | **0.964** | **3.875±1.026** | **3.865±0.926** | **0.964** | **_** | **_** | **_** |
|  | **HDL-C(mmol/L)** | **1.087±0.288** | **1.042±0.246** | **0.447** | **1.086±0.287** | **1.042±0.246** | **0.454** | **_** | **_** | **_** |
|  | **LDL-C(mmol/L)** | **2.344±0.812** | **2.411±0.785** | **0.689** | **2.346±0.81** | **2.411±0.785** | **0.693** | **_** | **_** | **_** |
|  | **APOA1(mmol/L)** | **1.18±0.252** | **1.174±0.206** | **0.901** | **1.18±0.251** | **1.174±0.206** | **0.902** | **_** | **_** | **_** |
|  | **APOB(mmol/L)** | **0.83±0.25** | **0.806±0.262** | **0.653** | **0.829±0.25** | **0.806±0.262** | **0.658** | **_** | **_** | **_** |
|  | **Lpa(mg/L)** | **246.421±252.709** | **220.98±162.486** | **0.62** | **245.599±250.104** | **220.98±162.486** | **0.625** | **_** | **_** | **_** |
|  | **NonHDLC(mmol/L)** | **2.773±1.057** | **2.823±0.988** | **0.818** | **2.774±1.053** | **2.823±0.988** | **0.821** | **_** | **_** | **_** |
| **Uyghur** |  | **CC(n=326)** | **AA+CA(n=47)** | **P** | **C（n=696）** | **A（n=50）** | **P** | **CA(n=44)** | **AA+CC(n=329)** | **P** |
|  | **TG（mmol/L)** | **1.917±1.318** | **2.276±1.525** | **0.091** | **1.932±1.32** | **2.373±1.64** | **0.027** | **2.164±1.372** | **1.935±1.345** | **0.294** |
|  | **TC(mmol/L)** | **4.015±1.108** | **4.532±1.341** | **0.016** | **4.049±1.132** | **4.493±1.314** | **0.025** | **4.576±1.37** | **4.013±1.104** | **0.013** |
|  | **HDL-C(mmol/L)** | **0.974±0.266** | **1±0.247** | **0.54** | **0.976±0.264** | **0.991±0.252** | **0.7** | **1.009±0.241** | **0.973±0.267** | **0.4** |
|  | **LDL-C(mmol/L)** | **2.564±0.847** | **3.023±1.159** | **0.012** | **2.597±0.877** | **2.952±1.158** | **0.041** | **3.105±1.154** | **2.557±0.846** | **0.004** |
|  | **APOA1(mmol/L)** | **1.095±0.277** | **1.12±0.217** | **0.55** | **1.096±0.274** | **1.126±0.211** | **0.459** | **1.114±0.222** | **1.096±0.276** | **0.674** |
|  | **APOB(mmol/L)** | **0.881±0.262** | **1.007±0.311** | **0.011** | **0.89±0.267** | **0.997±0.309** | **0.007** | **1.018±0.312** | **0.88±0.262** | **0.002** |
|  | **Lpa(mg/L)** | **244.831±240.482** | **346.63±430.509** | **0.123** | **251.587±258.554** | **344.557±423.315** | **0.136** | **348.992±438.574** | **245.482±241.135** | **0.136** |
|  | **NonHDLC(mmol/L)** | **0.058±0.235** | **0.174±0.383** | **0.052** | **3.068±1.103** | **3.432±1.378** | **0.074** | **0.186±0.394** | **0.058±0.234** | **0.042** |

Independent sample t test or ANOVA was conducted to generate the P values.The Hardy-Weinberg equilibrium test was performed by Chi-square test, SNP2 genotypes in Han(P=0.844) and Uyghur(P= 0.534) populations corresponded to Hardy-Weinberg equilibrium.

Abbreviation: TC:total cholesterol; TG:triglycerides; HDL-C:high-density lipoprotein cholesterol; LDL-C:low-density lipoprotein cholesterol; APOA1:apolipoprotein A1; APOB:apolipoprotein B,; Lpa:lipoprotein a; ALT:alanine aminotransferase.
