# Supplementary material for: Association of HMGCR rs17671591 and rs3761740 with lipidemia and statin response in Uyghurs and Han Chinese
Source: PeerJ. 2024 Sep 27;12:e18144. doi: 10.7717/peerj.18144 (PMC11441381; doi:10.7717/peerj.18144)
Supplement: Supplemental Information 9 — Chi-square test was conducted to generate the P values.The Hardy-Weinberg equilibrium test was performed by Chi-square test, SNP2 genotypes in Han(P=0.844) and Uyghur(P= 0.534) populations corresponded to Hardy-Weinberg equilibrium.Abbreviation: TC:total cholesterol; TG:triglycerides; HDL-C:high-density lipoprotein cholesterol; LDL-C:low-density lipoprotein cholesterol; APOA1:apolipoprotein A1; APOB:apolipoprotein B,; Lpa:lipoprotein a; ALT:alanine aminotransferase. [file peerj-12-18144-s009.docx]

**Table S7 Association of SNP2（rs3761740）with the rate of hyperlipidemia before oral statin**

| **Ethnic Group** |  | **Genotypes** | | | | **Allele** | | | | **Additive model** | | |
| --- | --- | --- | --- | --- | --- | --- | --- | --- | --- | --- | --- | --- |
| **Han** |  | **CC(n=380)** | **CA(n=25)** | **P** | **C（n=785）** | | **A（n=25）** | **P** | **_** | | **_** | **_** |
|  | High TG（%） | 31.152 | 28 | 0.741 | 31.052 | | 28..000 | 0.745 | _ | | _ | _ |
|  | High TC（%） | 12.565 | 16 | 0.85 | 12.674 | | 16 | 0.624 | _ | | _ | _ |
|  | Low HDL-C（%） | 39.267 | 48 | 0.387 | 39.544 | | 48 | 0.395 | _ | | _ | _ |
|  | High LDL-C（%） | 19.634 | 28 | 0.312 | 19.899 | | 28 | 0.32 | _ | | _ | _ |
|  | Low APOA1（%） | 6.053 | 12 | 0.569 | 6.242 | | 12 | 0.248 | _ | | _ | _ |
|  | High APOB（%） | 44.737 | 40 | 0.644 | 44.586 | | 40 | 0.65 | _ | | _ | _ |
|  | High Lpa（%） | 21.693 | 28 | 0.462 | 21.895 | | 28 | 0.469 | _ | | _ | _ |
|  | High Non-HDL-C（%） | 17.539 | 20 | 0.967 | 17.617 | | 20 | 0.759 | _ | | _ | _ |
| **Uyghur** |  | **CC(n=326)** | **AA+CA(n=47)** | **P** | **C（n=696）** | | **A（n=50）** | **P** | **CA(n=44)** | | **AA+CC(n=329)** | **P** |
|  | High TG（%） | 34.663 | 29.787 | 0.51 | 34.195 | | 32 | 0.752 | 27.273 | | 34.954 | 0.313 |
|  | High TC（%） | 7.975 | 27.66 | **＜0.001** | 9.339 | | 26 | **＜0.001** | 29.545 | | 7.903 | **＜0.001** |
|  | Low HDL-C（%） | 62.883 | 51.064 | 0.12 | 62.069 | | 52 | 0.158 | 50 | | 62.918 | 0.098 |
|  | High LDL-C（%） | 10.123 | 25.532 | **0.002** | 11.207 | | 24 | **0.007** | 27.273 | | 10.03 | **0.001** |
|  | Low APOA1（%） | 3.406 | 2.128 | 0.983 | 3.333 | | 2 | 0.92 | 2.273 | | 3.374 | 1 |
|  | High APOB（%） | 39.319 | 46.809 | 0.328 | 39.855 | | 46 | 0.392 | 47.727 | | 39.264 | 0.283 |
|  | High Lpa（%） | 27.019 | 40.426 | 0.058 | 27.826 | | 40 | 0.066 | 40.909 | | 27.077 | 0.057 |
|  | High Non-HDL-C（%） | 12.27 | 29.787 | **0.001** | 13.506 | | 28 | **0.005** | 31.818 | | 12.158 | **0.001** |

Chi-square test was conducted to generate the P values.The Hardy-Weinberg equilibrium test was performed by Chi-square test, SNP2 genotypes in Han(P=0.844) and Uyghur(P= 0.534) populations corresponded to Hardy-Weinberg equilibrium.

Abbreviation: TC:total cholesterol; TG:triglycerides; HDL-C:high-density lipoprotein cholesterol; LDL-C:low-density lipoprotein cholesterol; APOA1:apolipoprotein A1; APOB:apolipoprotein B,; Lpa:lipoprotein a; ALT:alanine aminotransferase.
