# Supplementary material for: Association of HMGCR rs17671591 and rs3761740 with lipidemia and statin response in Uyghurs and Han Chinese
Source: PeerJ. 2024 Sep 27;12:e18144. doi: 10.7717/peerj.18144 (PMC11441381; doi:10.7717/peerj.18144)
Supplement: Supplemental Information 10 — Chi-square test was conducted to generate the P values.The Hardy-Weinberg equilibrium test was performed by Chi-square test, SNP2 genotypes in Han(P=0.844) and Uyghur(P= 0.534) populations corresponded to Hardy-Weinberg equilibrium.Abbreviation: TC:total cholesterol; TG:triglycerides; HDL-C:high-density lipoprotein cholesterol; LDL-C:low-density lipoprotein cholesterol; APOA1:apolipoprotein A1; APOB:apolipoprotein B,; Lpa:lipoprotein a; ALT:alanine aminotransferase. [file peerj-12-18144-s010.docx]

**Table S8 Association of SNP2（rs3761740）with the rate of hyperlipidemia after oral statin**

|  |  | **Genotypes** | | | **Allele** | | | **Additive model** | | |
| --- | --- | --- | --- | --- | --- | --- | --- | --- | --- | --- |
| **HAN** |  | **CC(n=380)** | **CA(n=25)** | **P** | **C（n=785）** | **A（n=25）** | **P** | **CA** | **CC+AA** | **P** |
|  | High TG(%) | 22.632 | 24.000 | 0.874 | 22.675 | 24.000 | 0.876 | _ | _ | _ |
|  | High TC(%) | 3.684 | 4.000 | 0.935 | 3.694 | 4.000 | 0.936 | _ | _ | _ |
|  | Low HDL-C(%) | 45.000 | 56.000 | 0.285 | 45.350 | 56.000 | 0.293 | _ | _ | _ |
|  | High LDL-C(%) | 3.158 | 4.000 | 0.817 | 3.185 | 4.000 | 0.820 | _ | _ | _ |
|  | Low APOA1(%) | 53.476 | 52.000 | 0.886 | 53.428 | 52.000 | 0.888 | _ | _ | _ |
|  | High APOB(%) | 12.567 | 12.000 | 0.934 | 12.549 | 12.000 | 0.935 | _ | _ | _ |
|  | High Lpa(%) | 24.599 | 32.000 | 0.408 | 24.838 | 32.000 | 0.416 | _ | _ | _ |
|  | High NonHDLC(%) | 5.263 | 4.000 | 0.783 | 5.223 | 4.000 | 0.786 | _ | _ | _ |
| **Uyghur** |  | **CC(n=326)** | **AA+CA(n=47)** | **P** | **C（n=696）** | **A（n=50）** | **P** | **CA(n=44)** | **AA+CC(n=329)** | **P** |
|  | High TG(%) | 24.540 | 32.609 | 0.240 | 24.892 | 34.694 | 0.128 | 30.233 | 24.924 | 0.453 |
|  | High TC(%) | 3.988 | 13.043 | 0.009 | 4.604 | 12.245 | 0.019 | 13.953 | 3.951 | 0.005 |
|  | Low HDL-C(%) | 61.963 | 45.652 | 0.035 | 60.863 | 46.939 | 0.055 | 44.186 | 62.006 | 0.025 |
|  | High LDL-C(%) | 4.294 | 19.565 | ＜0.001 | 5.324 | 18.367 | ＜0.001 | 20.930 | 4.255 | 0.000 |
|  | Low APOA1(%) | 73.312 | 67.391 | 0.401 | 73.083 | 65.306 | 0.239 | 69.767 | 72.930 | 0.663 |
|  | High APOB(%) | 18.650 | 39.130 | 0.002 | 20.000 | 38.776 | 0.002 | 39.535 | 18.790 | 0.002 |
|  | High Lpa(%) | 26.452 | 34.783 | 0.238 | 26.998 | 34.694 | 0.245 | 34.884 | 26.518 | 0.249 |
|  | High NonHDLC(%) | 5.828 | 17.391 | 0.005 | 6.619 | 16.327 | 0.011 | 18.605 | 5.775 | 0.002 |

Chi-square test was conducted to generate the P values.The Hardy-Weinberg equilibrium test was performed by Chi-square test, SNP2 genotypes in Han(P=0.844) and Uyghur(P= 0.534) populations corresponded to Hardy-Weinberg equilibrium.

Abbreviation: TC:total cholesterol; TG:triglycerides; HDL-C:high-density lipoprotein cholesterol; LDL-C:low-density lipoprotein cholesterol; APOA1:apolipoprotein A1; APOB:apolipoprotein B,; Lpa:lipoprotein a; ALT:alanine aminotransferase.
