# Supplementary material for: Association of HMGCR rs17671591 and rs3761740 with lipidemia and statin response in Uyghurs and Han Chinese
Source: PeerJ. 2024 Sep 27;12:e18144. doi: 10.7717/peerj.18144 (PMC11441381; doi:10.7717/peerj.18144)
Supplement: Supplemental Information 11 — Independent sample t test or ANOVA was conducted to generate the P values.changes of lipids(TC, TG, HDL-C,LDL-C, APOA1, APOB,Lpa,non-HDL-C)=Lipids before oral statin-Lipid after oral statin)/Lipids before oral statin.Abbreviation: TC:total cholesterol; TG:triglycerides; HDL-C:high-density lipoprotein cholesterol; LDL-C:low-density lipoprotein cholesterol; APOA1:apolipoprotein A1; APOB:apolipoprotein B,; Lpa:lipoprotein a; ALT:alanine aminotransferase. [file peerj-12-18144-s011.docx]

Table S9 Association of SNP2（rs3761740）with changes of lipids after statin oral statin

|  |  | **Genotypes** | | | **Allele** | | | **Additive** | | |
| --- | --- | --- | --- | --- | --- | --- | --- | --- | --- | --- |
| Han |  | **CC(380)** | **CA(25)** | **P** | **C（785）** | **A（25）** | **P** | **CA(44)** | **AA+CC(329)** | **P** |
|  | changes of TG(%) | -3.491±55.011 | -2.303±50.859 | 0.916 | -3.453±54.816 | -2.303±50.859 | 0.918 | _ | _ | _ |
|  | changes of TC(%) | -23.973±19.732 | -21.029±24.803 | 0.478 | -23.878±19.891 | -21.029±24.803 | 0.485 | _ | _ | _ |
|  | changes of HDL-C(%) | 0.09±24.154 | -0.276±25.515 | 0.942 | 0.078±24.166 | -0.276±25.515 | 0.943 | _ | _ | _ |
|  | changes of LDL-C(%) | -32.39±21.858 | -27.759±31.939 | 0.322 | -32.241±22.228 | -27.759±31.939 | 0.329 | _ | _ | _ |
|  | changes of APOA1(%) | -2.564±21.768 | -4.298±14.095 | 0.695 | -0.06±0.301 | -0.073±0.221 | 0.837 | _ | _ | _ |
|  | changes of APOB(%) | -22.148±24.693 | -18.595±32.723 | 0.496 | -22.032±24.959 | -18.595±32.723 | 0.503 | _ | _ | _ |
|  | changes of Lpa(%) | 66.51±658.993 | -9.002±40.664 | 0.568 | 64.055±647.921 | -9.002±40.664 | 0.573 | _ | _ | _ |
|  | Changes of Non-HDL-C(%) | -28.9483±33.2084 | -24.6133±36.0891 | 0.53 | -28.8109±33.2664 | -24.6133±36.0891 | 0.536 | _ | _ | _ |
| Ughur |  | **CC(326)** | **AA+CA(47)** | **P** | **C（696）** | **A（50）** | **P** | **CA(44)** | **AA+CC(329)** | **P** |
|  | changes of TG(%) | -3.298±54.521 | 5.896±44.392 | 0.276 | -2.848±53.733 | 7.895±46.637 | 0.174 | 3.618±41.558 | -2.895±54.786 | 0.454 |
|  | changes of TC(%) | -13.865±32.355 | -12.161±22.967 | 0.731 | -13.776±31.820 | -11.868±22.415 | 0.68 | -12.494±23.577 | -13.803±32.221 | 0.797 |
|  | changes of HDL-C(%) | 7.535±39.311 | 6.824±32.567 | 0.907 | 7.517±38.871 | 6.441±32.328 | 0.85 | 7.261±32.833 | 7.468±39.223 | 0.974 |
|  | changes of LDL-C(%) | -18.343±32.453 | -14.786±29.001 | 0.483 | -18.027±32.246 | -15.919±28.496 | 0.657 | -13.496±29.513 | -18.486±32.337 | 0.339 |
|  | changes of APOA1(%) | -0.425±27.218 | -0.995±19.043 | 0.891 | -0.585±26.698 | 0.665±19.617 | 0.748 | -2.887±18.176 | -0.170±27.215 | 0.526 |
|  | changes of APOB(%) | -11.788±35.196 | -2.407±49.261 | 0.113 | -11.098±36.418 | -3.419±48.059 | 0.165 | -1.254±50.576 | -11.857±35.073 | 0.081 |
|  | changes of Lpa(%) | 147.068±1645.312 | 22.974±86.784 | 0.61 | 139.100±1589.622 | 20.918±84.678 | 0.603 | 25.317±89.071 | 145.542±1637.381 | 0.631 |
|  | Changes of Non-HDL-C(%) | -16.541±43.828 | -17.457±29.423 | 0.89 | -16.632±43.036 | -16.996±28.952 | 0.953 | -17.981±29.942 | -16.479±43.670 | 0.825 |

Independent sample t test or ANOVA was conducted to generate the P values.

changes of lipids(TC, TG, HDL-C,LDL-C, APOA1, APOB,Lpa,non-HDL-C)=Lipids before oral statin-Lipid after oral statin)/Lipids before oral statin.

Abbreviation: TC:total cholesterol; TG:triglycerides; HDL-C:high-density lipoprotein cholesterol; LDL-C:low-density lipoprotein cholesterol; APOA1:apolipoprotein A1; APOB:apolipoprotein B,; Lpa:lipoprotein a; ALT:alanine aminotransferase.
