# Supplementary material for: Association of HMGCR rs17671591 and rs3761740 with lipidemia and statin response in Uyghurs and Han Chinese
Source: PeerJ. 2024 Sep 27;12:e18144. doi: 10.7717/peerj.18144 (PMC11441381; doi:10.7717/peerj.18144)
Supplement: Supplemental Information 12 — Independent sample t test or ANOVA was conducted to generate the P values.changes of lipids(TC, TG, HDL-C,LDL-C, APOA1, APOB,Lpa,non-HDL-C)=Lipids before oral statin-Lipid after oral statin)/Lipids before oral statin.Abbreviation: TC:total cholesterol; TG:triglycerides; HDL-C:high-density lipoprotein cholesterol; LDL-C:low-density lipoprotein cholesterol; APOA1:apolipoprotein A1; APOB:apolipoprotein B,; Lpa:lipoprotein a; ALT:alanine aminotransferase. [file peerj-12-18144-s012.docx]

**Table S10 Association of SNP1（rs17671591）with change of lipids after oral statin**

|  |  | Dominant model | | | | Recessive model | | | | Additive model | | | Genotypes | | | | | | Allele | | |
| --- | --- | --- | --- | --- | --- | --- | --- | --- | --- | --- | --- | --- | --- | --- | --- | --- | --- | --- | --- | --- | --- |
| **H**  **a**  **n** |  | CC  (n=179) | TT+CT  (n=226) | P | TT  (n=36) | | CC+CT  (n=369) | P | CT  (n=190) | | CC+TT  (n=215) | P | CC  (n=179) | | CT  (n=190) | | TT  (n=36) | P | C  (n=548) | T  (n=262) | P |
|  | rate of changes of TG(%) | -5.658±  54.173 | -1.612±  55.179 | 0.463 | 2.878±  51.893 | | -3.984±  54.979 | 0.491 | -2.400±  55.830 | | -4.323±  53.792 | 0.726 | -5.658±  54.173 | | -2.400±  55.830 | | 2.878±  51.893 | 0.671 | -4.532±  54.673 | -1.028±  54.685 | 0.399 |
|  | rate of changes of TC(%) | -24.435±  17.934 | -23.268±  21.652 | 0.564 | -22.952±  17.155 | | -23.864±  20.323 | 0.803 | -23.324±  22.386 | | -24.203±  17.783 | 0.663 | -24.435±  17.934 | | -23.324±  22.386 | | -22.952±  17.155 | 0.843 | -24.051±  19.559 | -23.227±  21.092 | 0.589 |
|  | rate of changes of HDL-C(%) | -0.767±  23.578 | 0.738±  24.737 | 0.538 | -1.639±  22.322 | | 0.22±  24.393 | 0.673 | 1.156±  25.168 | | -0.904±  23.336 | 0.397 | -0.767±  23.578 | | 1.156±  25.168 | | -1.639±  22.322 | 0.686 | -0.103±  24.112 | 0.429±  24.408 | 0.772 |
|  | rate of changes of LDL-C(%) | -32.514±  20.493 | -31.767±  24.197 | 0.743 | -30.784±  18.580 | | -32.219±  22.94 | 0.727 | -31.940±  25.090 | | -32.243±  20.174 | 0.894 | -32.514±  20.493 | | -31.940±  25.090 | | -30.784±  18.580 | 0.914 | -32.315±  22.151 | -31.639±  23.513 | 0.694 |
|  | rate of changes of APOA1  (%) | -0.368±  21.750 | -4.528±  20.904 | 0.053 | -8.063±  19.151 | | -2.186±  21.505 | 0.13 | -3.908±  21.184 | | -1.571±  21.505 | 0.276 | -0.368±  21.750 | | -3.908±  21.184 | | -8.063±  19.151 | 0.090* | -1.591±  21.582 | -4.988±  20.683 | **0.036** |
|  | rate of changes of APOB(%) | -22.582±  23.013 | -21.395±  26.933 | 0.642 | -17.211±  30.382 | | -22.351±  24.722 | 0.263 | -22.133±  26.299 | | -21.738±  24.313 | 0.876 | -22.582±  23.013 | | -22.133±  26.299 | | -17.211±  30.382 | 0.527 | -22.427±  24.155 | -20.849±  27.379 | 0.412 |
|  | rate of changes of Lpa(%) | 107.07±  945.526 | 25.295±  122.469 | 0.205 | 38.988±  146.232 | | 63.818±  665.119 | 0.831 | 22.879±  118.075 | | 96.372±  869.914 | 0.253 | 107.071±  945.526 | | 22.879±  118.075 | | 38.988±  146.232 | 0.444 | 77.969±  767.578 | 27.081±  125.584 | 0.295 |
|  | rate of changes of NonHDLC  (%) | -30.667±  24.193 | -27.108±  39.117 | 0.286 | -22.283±  32.073 | | -29.303±  33.460 | 0.228 | -28.018±  40.316 | | -29.269±  25.780 | 0.706 | -30.667±  24.194 | | -28.018±  40.316 | | -22.283±  32.073 | 0.362 | -29.749±  30.726 | -26.448±  38.211 | 0.187 |
| **U**  **y**  **g**  **h**  **u**  **r** |  | CC  (n=142) | TT+CT  (n=231) | P | TT  (n=56) | | CC+CT  (n=317) | P | CT  (n=175) | | CC+TT  (n=198) | P | CC  (n=142) | | CT  (n=175) | | TT  (n=56) | P | C  (n=459) | T  (n=287) | P |
|  | rate of changes of TG(%) | 2.648±  64.513 | -5.059±  44.965 | 0.186 | -1.994±  48.459 | | -2.126±  54.221 | 0.987 | -6.013±  43.931 | | 1.364±  60.410 | 0.194 | 2.648±  64.513 | | -6.013±  43.931 | | -1.994±  48.459 | 0.373 | -0.647±  57.573 | -4.471±  45.580 | 0.354 |
|  | rate of changes of TC(%) | -13.969±  32.712 | -13.443±  30.425 | 0.878 | -17.055±  32.120 | | -13.059±  31.146 | 0.395 | -12.318±  29.888 | | -14.823±  32.493 | 0.452 | -13.969±  32.712 | | -12.318±  29.888 | | -17.055±  32.120 | 0.628 | -13.341±  31.605 | -14.136±  30.729 | 0.742 |
|  | rate of changes of HDL-C(%) | 10.829±  44.151 | -5.341±  34.404 | 0.192 | 3.068±  28.154 | | 8.194±  39.956 | 0.375 | 6.048±  36.177 | | 8.682±  40.442 | 0.52 | 10.829±  44.151 | | 6.048±  36.177 | | 3.068±  28.154 | 0.379 | 9.010±  41.268 | 4.904±  33.260 | 0.167 |
|  | rate of changes of LDL-C(%) | -20.640±  29.200 | -16.980±  33.740 | 0.285 | -25.320±  27.070 | | -17.16±  32.760 | 0.079 | -14.292±  35.271 | | -21.964±  28.624 | **0.021** | -20.641±  29.200 | | -14.292±  35.271 | | -18.375±  32.092 | **0.046** | -17.673±  31.601 | -18.220±  32.660 | 0.825 |
|  | rate of changes of APOA1  (%) | -2.842±  32.07 | -2.573±  21.757 | 0.059 | -1.297±  29.148 | | -0.362±  25.807 | 0.813 | -2.970±  18.972 | | 1.697±  31.267 | 0.095* | 2.842±  32.070 | | -2.970±  18.972 | | -1.297±  29.148 | 0.156 | 0.631±  27.900 | -2.328±  23.301 | 0.145 |
|  | rate of changes of APOB(%) | -11.862±  36.742 | -9.762±  37.828 | 0.609 | -16.314±  26.604 | | -9.572±  38.887 | 0.23 | -7.710±  40.564 | | -13.100±  34.214 | 0.177 | -11.862±  36.742 | | -7.710±  40.564 | | -16.314±  26.604 | 0.308 | -10.281±  38.209 | -11.024±  35.990 | 0.798 |
|  | rate of changes of Lpa(%) | 107.568±  924.263 | -145.344±  1814.842 | 0.823 | 18.614±  67.525 | | 150.295±  61.637 | 0.569 | 185.043±  2079.326 | | 82.832±  786.310 | 0.533 | 107.568±  924.263 | | 185.043±  2079.326 | | 18.614±  67.525 | 0.774 | 137.066±  1472.314 | 120.937±  1631.052 | 0.892 |
|  | rate of changes of NonHDLC(%) | -18.152±  43.255 | -15.737±  41.694 | 0.593 | -18.850±  52.708 | | -16.2689±  40.214 | 0.674 | -14.741±  37.624 | | -18.349±  45.987 | 0.411 | -18.152±  43.255 | -14.741±  37.624 | | -18.850±  52.708 | | 0.71 | -16.851±  41.140 | -16.345±  43.975 | 0.873 |

Independent sample t test or ANOVA was conducted to generate the P values.

changes of lipids(TC, TG, HDL-C,LDL-C, APOA1, APOB,Lpa,non-HDL-C)=Lipids before oral statin-Lipid after oral statin)/Lipids before oral statin.

Abbreviation: TC:total cholesterol; TG:triglycerides; HDL-C:high-density lipoprotein cholesterol; LDL-C:low-density lipoprotein cholesterol; APOA1:apolipoprotein A1; APOB:apolipoprotein B,; Lpa:lipoprotein a; ALT:alanine aminotransferase.
